# Primary Postnatal Dorsal Root Ganglion Culture from Conventionally Slaughtered Calves

**DOI:** 10.1371/journal.pone.0168228

**Published:** 2016-12-09

**Authors:** A. Fadda, M. Bärtschi, A. Hemphill, H. R. Widmer, A. Zurbriggen, P. Perona, B. Vidondo, A. Oevermann

**Affiliations:** 1 Division of Neurological Sciences, Vetsuisse Faculty, University of Bern, Bern, Switzerland; 2 Graduate School for Cellular and Biomedical Sciences, Theodor Kocher Institute, University of Bern, Switzerland; 3 Institute for Parasitology, Vetsuisse Faculty, University of Bern, Bern, Switzerland; 4 Neurocenter and Regenerative Neuroscience Cluster, University Hospital and University of Bern, Bern, Switzerland; 5 School of Engineering, The University of Edinburgh, Edinburgh, United Kingdom; 6 Veterinary Public Health Institute (VPHI), Vetsuisse Faculty, University of Bern, Bern, Switzerland; National Eye Centre, UNITED STATES

## Abstract

Neurological disorders in ruminants have an important impact on veterinary health, but very few host-specific *in vitro* models have been established to study diseases affecting the nervous system. Here we describe a primary neuronal dorsal root ganglia (DRG) culture derived from calves after being conventionally slaughtered for food consumption. The study focuses on the *in vitro* characterization of bovine DRG cell populations by immunofluorescence analysis. The effects of various growth factors on neuron viability, neurite outgrowth and arborisation were evaluated by morphological analysis. Bovine DRG neurons are able to survive for more than 4 weeks in culture. GF supplementation is not required for neuronal survival and neurite outgrowth. However, exogenously added growth factors promote neurite outgrowth. DRG cultures from regularly slaughtered calves represent a promising and sustainable host specific model for the investigation of pain and neurological diseases in bovines.

## Introduction

Dorsal root ganglia (DRG), or spinal ganglia are isolated nodular thickenings of the dorsal spinal nerve roots, which contain somas of afferent sensory neurons carrying nociceptive, mechanoceptive and thermoceptive signals from the periphery to the central nervous system (CNS). These neurons are of pseudo-unipolar type, and their somas are tightly insulated by satellite cells [1–3].

Due to their easy identification and isolation, DRG cultures are widely used to study molecular mechanisms of neuropathic and inflammatory pain and to evaluate pharmacological and toxic effects of substances on neuronal survival and growth [4–7]. Furthermore, DRG cultures are used as models for neurite outgrowth and synapse formation during development and for studies on post-injury axonal regeneration [7–9]. Recent studies have demonstrated the stem cell potential of satellite cells, and used DRGs to analyze neurogenesis in the peripheral nervous system [10]. Finally, because DRG sensory neurons project fibers that connect the periphery to the CNS these cultures have been used to investigate the intra-axonal spread of pathogens to the CNS [6, 11–13].

Generally, DRG cultures originate from embryonic, neonatal or adult mice and rats, whilst neuronal cultures from other species have been rarely employed [14–19]. Here, we describe the culture of primary postnatal DRG neurons derived from calves that were slaughtered for food consumption. We investigated neuronal survival and growth of non-neuronal cells over time and analyzed the effects of selected growth factors on neuronal viability, neurite outgrowth and arborisation. Our results show that postnatal bovine DRG neurons survive in mixed cell cultures for extended periods of time and establish an intricate neurite network, irrespective of growth factor supplementation. This DRG culture may represent a promising and sustainable host-specific *in vitro* model for the study of the pathophysiology of pain and toxic neuronal injury and may additionally be used as model for neurological diseases in ruminants.

## Materials and Methods

### Animals and dorsal root ganglia

Dorsal root ganglia from calves, not more than 6 months of age, were collected at a small local slaughterhouse in the Canton Bern, Switzerland, immediately after conventional slaughtering. Animals were shot with a captive bolt pistol according to the local legislations. Carcasses were divided into halves along the spine, and cervical, thoracic and lumbar DRGs were isolated from the intervertebral foramina of the opened vertebral canal by dissecting the dorsal root from the spinal cord and the spinal nerve immediately distal to the ganglion with sterile forceps and scissors. Minimum 24 DRG were pooled from each carcass and immediately immersed in ice-cold HBSS (Hank's Balanced Salt Solution-Sigma Aldrich, Switzerland). This procedure required approximately 20 minutes. Samples were transported to the laboratory within 25 minutes. DRGs from two different animals were immersion-fixed in 10% neutral-buffered formalin for 48h and embedded in paraffin. Sections of the paraffin embedded tissue samples were cut at 5-μm thickness, mounted on adhesive glass slides and routinely stained with hematoxylin and eosin (HE) for morphological examination.

### Dissociation of DRG and cell culture

Dissociation of DRGs was performed as previously described in other species with slight modifications [5, 6, 12]. Non-nervous tissue and nerve roots were carefully removed from each ganglion under the laminar flow hood using sterile instruments. The ganglia were cut into small pieces (approximately 1–2 mm side length) and added to a dissociation solution consisting of HBSS supplemented with collagenase (at a concentration of 500 UI/ml; collagenase from *Clostridium histolyticum* C5138, Sigma Aldrich, Switzerland), hyaluronidase (at a concentration of 150 UI/mL; hyaluronidase Type IV Sigma Aldrich, Switzerland) and 3 μl/ml of 3.0 M CaCl^2^ solution. DRGs were incubated in the dissociation solution (approximately 4 ml per 6 DRGs) at 37° for a total of 3 hours. During the digestion, the DRGs were triturated three times by gently pipetting the tissue up and down for 10 minutes (~30 times) at 60 minute intervals. For trituration, a 10 ml polystyrene serological pipet (Falcon^™^, Thermo Fisher, AG Reinach, Switzerland) with a tip opening size of 1.5 mm was used. The digestion process was stopped by adding an equal volume of HBSS, and the suspension was filtered through a stainless mesh sieve (pore size 104 μm) in order to decrease the amount of debris in the primary culture. The obtained cell suspension was centrifuged for 5 minutes (1000 G at 21°) and the supernatant was discarded. The residual pellet was double-layered with an upper white layer containing mainly non-neuronal elements and a lower yellow layer containing mainly neurons. The upper layer was carefully removed with a Pasteur pipette and discarded, while the underlying layer with neurons was resuspended in culture medium, composed of serum-free Neurobasal medium (NBM; Gibco; Thermo Fisher Scientific, Inc.) supplemented with B27 (10 μl/ml, Gibco; Thermo Fisher Scientific, Inc.) penicillin 100 UI/ml and streptomycin 100 μg/ ml (Gibco; Thermo Fisher Scientific, Inc.). Neurons were counted in a Nageotte cell counter chamber and used at a density of approx. 500 neurons per well in a 24 wells tissue culture plate (Vitaris, Baar, Switzerland), containing 12-mm coverslips that were previously coated with poly-L-ornithine hydrobromide (0.1mg/ml in HBSS overnight, Sigma Aldrich, Switzerland) and laminin (10 μg/ml in HBSS for 5h, Laminin from mouse Engelbreth-Holm-Swarm sarcoma, Roche Diagnostic, Switzerland). To evaluate for optimal culture conditions, DRG culture medium was supplemented with the exogenous growth factors GDNF (human glial derived nerve growth factor, SRP3309 Sigma Aldrich, Switzerland), IGF (human insulin-like growth factor-I, I3769 Sigma Aldrich, Switzerland) and NGF (Nerve Growth Factor-7S N0513 from murine submaxillary gland, Sigma Aldrich, Switzerland). Neuron viability, neurite outgrowth and arborisation were evaluated. These growth factors were supplemented either as a single formula in increasing concentrations (NGF: 10, 50, 150 ng/ml; GDNF: 10, 50, 150 ng/ml; IGF: 5, 20, 50 ng/ml) or as combined formulas (NGF 50 ng/ml combined with GDNF 50 ng/ml: COMBO2; NGF 50ng/ml combined with GDNF 50 ng/ml and IGF20 ng/ml: COMBO3). Controls consisted of DRGs cells maintained in culture medium without exogenous growth factor supplementation. Culture medium was replaced every 48 hours. At 24h, 3 days and 7 days, coverslips were harvested for characterization of neurons and non-neuronal cells. For growth factor analysis, five independent experiments consisting of duplicates were performed with DRG of five different animals. For identification of non-neuronal cells, three independent experiments with DRGs of three different animals were performed.

### Immunofluorescence

Coverslips were removed from the 24-wells plate and were fixed in 4% paraformaldehyde (PFA Sigma Aldrich) for 30 min at room temperature (RT). Next, coverslips were washed three times in 0.5% Tween phosphate-buffered saline (PBS-T), and cells were permeabilized with 0.5% Triton X-100 in PBS for 30 minutes at RT. To block non-specific labelling, cells were incubated in 10% normal goat serum (Dako, Baar, Switzerland) in PBS-T for 30 min. Primary antibodies including polyclonal rabbit anti-S100 (S100, Dako, Glostrup, Denmark 1:600), monoclonal mouse anti-vimentin (Vim 3b4, M7020, Dako, Glostrup, Denmark, 1:100), monoclonal mouse anti-neurofilament (M0762, Dako, Glostrup, Denmark, 1:100), polyclonal rabbit anti-βIII tubulin antibody (Ab18207, AbCam, Cambridge, UK, 1:500), polyclonal rabbit anti-GFAP (Glial fibrillary acidic protein, Z0334, Dako, Glostrup, Denmark, 1:1000), monoclonal mouse anti-E-cadherin (610181, purified mouse anti-human E-cadherin, BD Laboratories, Sparks, MD, USA 1:100) and polyclonal rabbit anti-periaxin (HPA001868, Sigma-Aldrich, St. Louis, MO, USA, 1:300) were applied for 1 hour at RT. For E-cadherin labeling heat induced antigen retrieval in Citrate buffer pH6 was performed as previously described [20]. Then, cells were washed with PBS-T 3 times and incubated with Alexa Fluor 555-conjugated goat anti-rabbit and Alexa Fluor 488-conjugated goat anti-mouse secondary antibodies (Life technologies Carlsbad, CA, USA 1:100) for one hour in the dark. Cells were then washed 3 times for 5 minutes with PBS-T and incubated with DAPI (Invitrogen, Carlsbad, CA, USA, T3604, 1:10000) for 45 minutes. Finally, coverslips were washed once with PBS-T, rinsed with distilled water, and mounted on Superfrost Plus glass slides (Menzel-Gläser) with glycerol mounting gel (Dako, Glostrup, Denmark). Cell cultures were imaged using an Olympus Fluoview FV1000 confocal microscope (Olympus, Tokyo, Japan), equipped with 405 nm, 488 nm, 555 nm and 633 nm laser channels.

### Transmission electron microscopy (TEM)

DRG cultures grown on glass coverslips were washed in 100 mM sodium cacodylate buffer (pH 7.3) and were fixed in 2.5% glutaraldehyde in cacodylate buffer for 2 hours at RT. They were then washed three times in cacodylate buffer and post-fixation was done in 2% osmium tetroxide in cacodylate buffer for 2 hours. Following several washes in water, specimens were pre-stainend in saturated uranyl acetate in water, washed and dehydrated in an ascending ethanol series (30-50-70-90 and three times 100%). They were then embedded in Epon 812 resin as described earlier [21]. Polymerization of the resin was carried out at 65°C overnight. The glass coverslip was removed from the Epon block by dipping it into liquid nitrogen, and sections were cut parallel to the adhesion surface using an ultramicrotome (Reichert Jung, Vienna, Austria). They were loaded onto 300-mesh copper grids (Plano GmbH, Marburg, Germany), and staining with uranyl acetate and lead citrate was performed as described [22].

### Viability assay

To evaluate the effects of growth factors on neuronal survival rates, coverslips of unsupplemented cultures, cultures supplemented with either NGF 50 ng/ml or with a combined formula consisting of NGF 50 ng/ml, GDNF 50 ng/ml and IGF 20 ng/ml (COMBO3) were immersed in a calcein-viability stain (calcein-AM, Sigma Aldrich) at a final concentration of 20 nM/well in PBS for 30 minutes at 37°C in a CO_2_ incubator. The number of stained neurons and the total number of neurons in ten fields of view (FOV) per coverslip were counted with the Olympus Fluoview FV1000 confocal microscope using a 10X objective. Neuron viability was expressed as the percentage of calcein-stained neurons compared to the total neuron number. Cell viability analysis was assessed at three different time points (week 1, 2, 3 of culture). Three independent experiments with tissues from three different animals were performed in triplicates.

### Morphological analysis of neurons

For morphological analysis of neurons, the Sholl profile was obtained of 90 neurons for each GF supplementation and control. Five independent experiments were performed with DRGs from five different calves. Neurons were identified by their large size and round and voluminous shape in bright-field microscopy, combined with a large, pale DAPI-staining nucleus and neurofilament (NF) expression. In order to validate the labeling of neuronal soma and neurites by the anti-NF antibody, coverslips from two different experiments were simultaneously labeled with the neuron-specific anti-βIII tubulin antibody and anti-NF antibody, and sixty neurons per condition of GF supplementation were checked for co-labelling.

Confocal zeta-stack images of neurons and their neurite arborisation area covering a depth of 20–100 μm were acquired with the Olympus Fluoview FV1000 confocal microscope (Olympus, Tokyo, Japan). Neuronal arborization was assessed with the Sholl analysis plug-in for the open source platform for biological image analysis FIJI (http://fiji.sc/Sholl_Analysis) [23–25] using 2D grayscale stack images of 90 isolated neurons per condition stained with the anti-NF antibody. To this end, 8-bit grayscale images of neurons were pre-processed by thresholding and removal of pixels representing debris, unspecific background staining or neurites from adjacent neurons in the case of neurite overlap.

**Fig 1:**
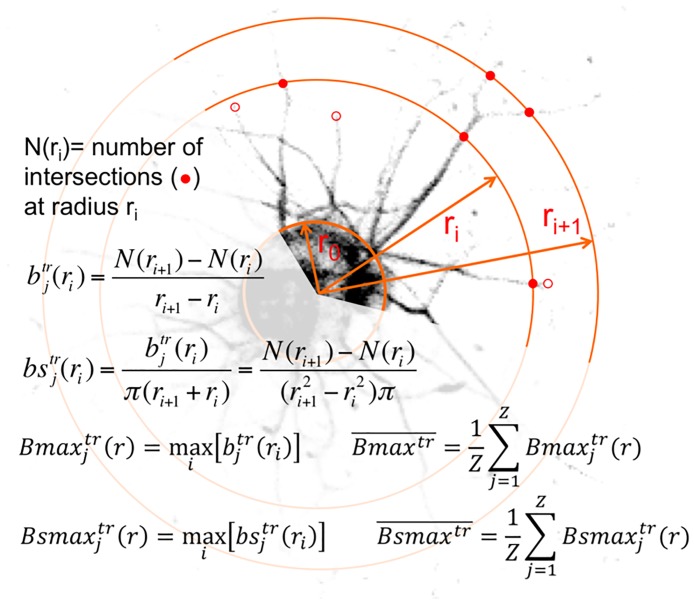
Definition of data used for the Sholl analysis and mathematical formulas used for the estimation of neurite branching based on the Sholl profiles. Red filled circles indicate intersections at the generic radius r_i_ and r_i+1_, whereas red empty circles indicate terminating neurites between two successive radii. (j) = neuron; (tr) = treatment; (i) = generic radius number; N(r_i_) = number of intersections at radius r_i_; N(r_i+1_) = number of intersections at radius r_i+1_; (b_j_^tr^(r_i_)) = branching rate; (bs_j_^tr^(r_i_) = specific branching; (Bmax_j_^tr^(r)) = maximum branching; (Bsmax_j_^tr^(r)) = maximum specific branching.

For each neuron a Sholl profile was obtained based on the number of neurites intersecting concentric circles of an overlaying grid at specified radial distances (Fig 1). For all neurons, the starting radius was set at 50 μm and incremental radial steps were set at 10 μm.

Additionally to the Sholl profile, the following parameters were computed: the enclosing radius, which is the radius of the most external circle of the Sholl grid intersected by neurites; the maximum number of intersections at one of all radii; the radius of maximum intersections, which describes the distance at which the maximum number of intersections was observed. Based on the Sholl profiles, the branching rate (*b*_*j*_^*tr*^(*r*_*i*_)) was estimated for 30 neurons *j* per treatment *tr* based on the number of intersections *N*(*r*) at radii *r*_i_ and r_i+1_ (Fig 1). Additionally, the maximum branching (*Bmax*_*j*_^*tr*^(*r*)) and minimum branching (*Bmin*_*j*_^*tr*^(*r*))) rates were defined by identifying the maximum and the minimum amongst the branching rates of all radii. By dividing the branching rate *b*_*j*_^*tr*^(*r*_*i*_) by the area between two consecutive radial circles (radii *r*_i_ and r_i+1_) the specific branching, *bs*_*j*_^*tr*^(*r*_*i*_) and their related maximum and minimum specific branching rates were obtained. Mean maximum and minimum branching rate, $\bar{Bmax^{tr}}$ and $\bar{Bmin^{tr}}$, and mean maximum and minimum specific branching, $\bar{Bsmax^{tr}}$ and $\bar{Bsmin^{tr}}$, were calculated by averaging the maximum and minimum values over the number of samples. Sholl profiles, enclosing radius, maximum number of intersections, radius of maximum intersections and branching rates were compared between the experimental groups.

### Statistical analysis and graphs

Statistics and graphical representation of the data were generated using Graph Pad Prism 5.0 (Graph Pad Software Inc., La Jolla, CA, USA) and MATLAB-The MathWorks program (Natick, Massachusetts, USA). Sholl and branching data distributions were assessed for Normality using Kolmogorov-Smirnoff tests, D’Agostino and Pearson omnibus normality tests, and Shapiro-Wilk tests. The differences in enclosing radius, maximum number of intersections, radius of maximum intersections and branching rates for the different experimental groups were assessed using a repeated-measures Anova or a non-parametric Kruskal-Wallis test (if the variable was not normally distributed), followed by post hoc correction with Dunn's multiple comparisons test. Pearson's correlation coefficient was calculated to determine correlation between minimum and maximum branching. The association between the presence of neurites and a soma diameter bigger or smaller than 50 microns was evaluated using a X2 test. P values lower than 0.05 were considered to indicate significant differences.

## Results

### Bovine dissociated DRG provide mixed cell cultures with neurons that emit neurites and can be maintained for extended period of time

**Fig 2:**
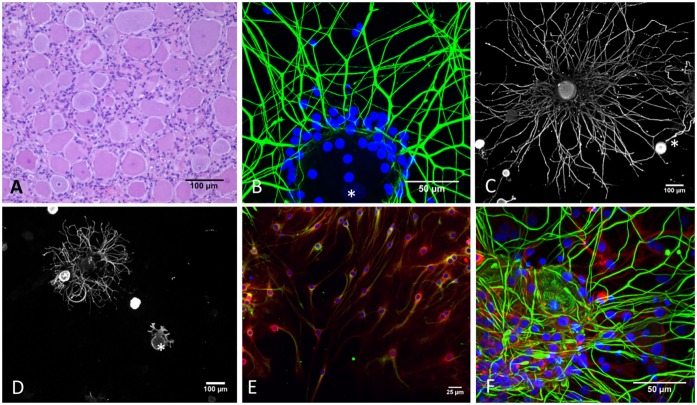
DRG neurons *in situ* and *in vitro*. (A) Histological section of a bovine DRG stained with hematoxylin and eosin (HE). Satellite cells tightly cluster around large sensory neurons. (B) Immunofluorescence staining of dissociated DRG at day 3 in culture, which contains a mixed population of cells. The neuron emits a large number of neurofilament-positive neurites (green). Satellite cells, whose nuclei are stained with DAPI (blue), remain tightly clustered around the soma of the dissociated neuron. (*) Weak DAPI-staining of the neuronal nucleus. (C) A large sensory DRG neuron, stained with neurofilament (grey), showing an extensive neurite outgrowth and few smaller neurons staining intensively with NF that have no detectable neurite outgrowth at 3 days of culture (*). (D) Neuron stained with neurofilament showing filopodia like structures (*) at day 1 in cell culture next to a neuron with neurite outgrowth and neurons without neurites. (E) DRG culture at day 7. Satellite/Schwann cells expressing S100 (red) and Vimentin (green) constituting a subconfluent layer at day 7 of culture. Nuclei are stained with DAPI (blue). (F) Immunofluorescence staining showing GFAP-positive satellite cells (red) surrounding a neuron labeled with neurofilament (green). Nuclei are stained with DAPI (blue).

In average, a yield of approximately 10.000 neurons per DRG was achieved. Analogous to DRG neurons *in situ* (Fig 2A), DRG neurons cultured *in vitro* largely varied in size. Neurons had large spherical somas ranging from 20 to 200 μm diameter (Fig 2B, 2C and 2D), and a centrally located nucleus that stained weakly with DAPI (Fig 2B). A number of neurons emanated a branching network of neurites that were labeled with anti-NF, whilst others attached to the coverslip but lacked neurites (Fig 2C and 2D) (S3 Fig). These results were confirmed in two experiments by double-immunofluorescence (IF) with anti-NF and anti-βIII tubulin antibodies, which revealed a high consistency between NF and βIII tubulin expression. Only a minimal number of neurons (1.6%) had neurites that were detected by the anti-βIII tubulin antibody but not by the anti-NF antibody. However, anti-βIII tubulin labeled terminal neurites more accurately. Twenty % (72/360) of the analyzed neurons exhibited a longer neurite length when stained with anti β-III tubulin antibodies.

**Fig 3:**
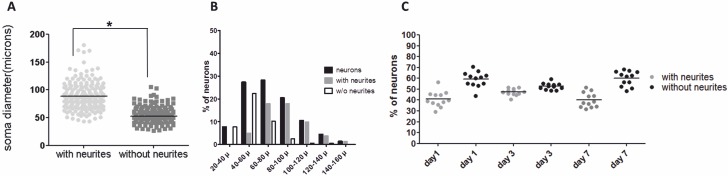
Ratio of neurite-bearing neurons and neurons without neurons. The soma diameter of 360 neurons was measured and the presence or absence of neurites was assessed. (A) Dot plots showing soma diameter of neurons with and without neurites. Black horizontal lines indicate the mean. Based on soma diameter two categories (large neurons >50 μm and small neurons <50 μm) were defined. The prevalence of neurites was significantly higher in the group of large neurons as in small neurons (*) (X2 test, p < 0.0001). (B) Frequency histogram showing the distribution of neurons with and without neurites in function of size. The frequency distribution of neurons with neurites (grey) is shifted to a larger soma diameter compared to neurons without neurites (white). (C) Dot plot showing the proportions of neurons with and without neurites over 7 days of culture. The proportion of neurons with and without neurites did not change significantly over the entire period of the experiment (Kruskal Wallis test, p > 0.05). Black horizontal lines indicate the mean.

The presence of neurites was significantly associated with large neurons (Fig 3A), but small-sized neurons with neurites were also observed. Neurons with a diameter smaller than 40 μm rarely emitted neurites, and the proportion of neurite bearing cells increased in larger sized neurons (Fig 3B). The proportion of neurons with and without neurites remained consistent over one week of culture (Fig 3C).

**Fig 4:**
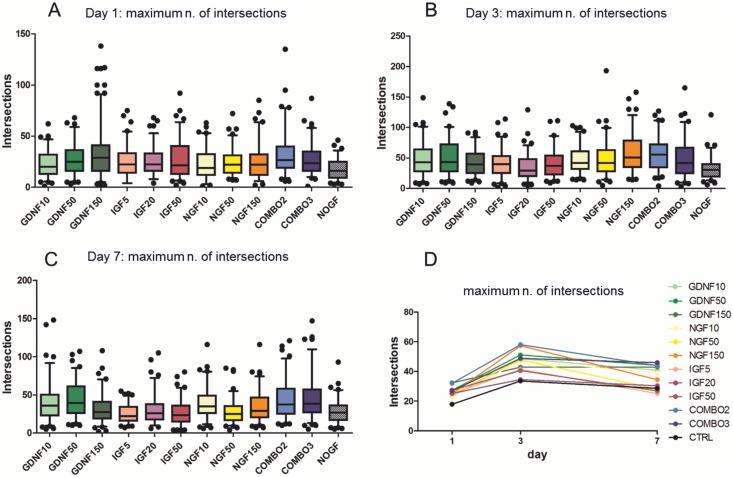
GF supplementation modulates but is not required for neurite outgrowth. (A, B, C) Box-and-whisker plots (5–95 percentiles) show the maximum number of intersections over the culture time period at day 1 (A), day 3 (B) and day 7 (C). Values are compared between GF (NGF: 10, 50, 150 ng/ml; GDNF: 10, 50, 150 ng/ml; IGF: 5, 20, 50 ng/ml, NGF 50 ng/ml combined with GDNF 50 ng/ml [COMBO2]; NGF 50ng/ml combined with GDNF 50 ng/ml and IGF20 ng/ml [COMBO3]) and control (No GF). Values below and above the whiskers are shown as individual points. Horizontal black lines indicate the median. Ninety neurons were analyzed for each GF supplementation and control. GDNF50, GDNF150, IGF20, IGF50, NGF50, COMBO2, COMBO3 induced a significant increase in the maximum number of intersections at day 1. At day 3 GDNF and NGF alone or in combined formulation but not IGF increased the maximum number of intersections. At day 7 both IGF and NGF 50 treated neurons showed a significant lower number of intersections. (p<0.05—Kruskall Wallis test, results are shown in S1 File). (D) Comparison of maximum number of intersections over time between supplementations and control. The number of intersections was higher in GF supplemented neurons compared to control and increased from day 1 to 3. From day 3 to day 7, the maximum number of intersections reached a plateau phase in all treatment groups. Mean values of maximum number of intersections calculated with the Sholl analysis are shown.

Prominent neurite outgrowth with occasional lamellipodia and filopodia-like cytoplasmic protrusions was obvious at day 1 of culture (Fig 2D). Neurite arborisation as assessed by the maximum number of intersections increased until day 3 of culture, but lamellipodia and filopodia-like cytoplasmatic protrusions became less obvious at this time point. Between day 3 and day 7 of *in vitro* culture, neurite arborisation reached a plateau (Fig 4D). In contrast, neurite length (enclosing radius) and radius of maximum number of intersections continued to increase until day 7 (S1 and S2 Figs).

**Fig 5:**
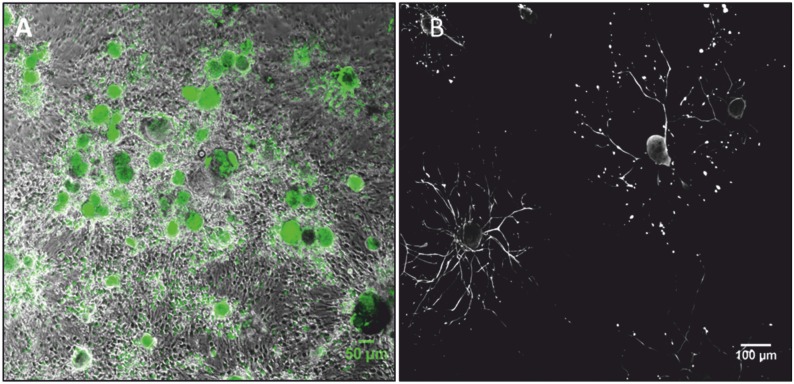
DRG neurons can be maintained in culture for extended periods. (A) Viable DRG neurons after 2 weeks in culture stained with calcein (green). (B) Diffuse neurite fragmentation in a neurofilament-positive neuron (*) at day 7. In the lower left a viable neurite bearing neuron is present.

Neurons were viable in culture for a minimum of 3 weeks (Fig 5A), and in long-term experiments they were viable for more than 4 weeks. Viability over time was similar between non-supplemented cultures and cultures treated with exogenously added growth factors (data not shown). Overall, the proportion of viable neurons at week 1 was 81.21% and slightly decreased at week 2 (79.74%) and 3 (73.04%). Corroborating these data, IF analysis at day 1 and 3 did not identify any morphological evidence of significant neuronal degeneration in cultures with or without growth factor supplementation. Only at day 7 very few neurons displayed an irregular outline of the soma, axonal beading and fragmentation (Fig 5B).

DRG cultures contained contaminating non-neuronal cells. Most cells were tightly clustered around the neuronal somata, similar to the capsule of satellite cells around DRG neurons observed *in situ* (Fig 2A and 2B, S6C Fig). In between neurons, two populations of cells were observed (Fig 2E). The majority of cells had long slender bipolar cytoplasmic processes and a central oval nucleus that expressed S100 protein, GFAP and vimentin indicating Satellite/Schwann cell origin. Additionally, TEM revealed the presence of cilia, exhibiting a characteristic microtubular cytoskeleton and extending from a ciliary pocket [26], confirming neuroectodermal origin (S6D Fig) [27, 28]. These cells followed the course of neurites and enclosed them. However, myelin formation around neurites was not observed by TEM (S6A and S6B Fig).

Membranous E-cadherin was expressed by satellite cells tightly clustered around the neuron. Periaxin expression was not observed in the glia cell population. The minority of contaminating non-neuronal cells had a flat, polygonal morphology and expressed vimentin only, which is reminiscent for fibroblasts. Contaminating cells proliferated and constituted generally a subconfluent layer at day 7 of culture.

### Growth factor supplementation modulates but is not required for neurite growth and branching

The proportion of viable neurons as well the proportion of neurons with and without neurites was not significantly different between the groups supplemented with growth factors and the control group (data not shown). Additionally, a correlation between the mean values of minimum and maximum branching rates was observed in all experimental groups at each time point (S5 Fig).

In contrast, supplementation with growth factors had an effect on neurite outgrowth and arborisation. In many growth factor treated cultures, the maximum number of intersections and enclosing radius were superior compared to the control from day 1 to day 7 of culture (Fig 4, S2 Fig).

When compared to non-supplemented controls, GDNF promoted neurite outgrowth and arborisation (Fig 4, S1 and S2 Figs, S1–S3 Files). At most time points, GDNF concentrations of 10ng/ml and 50ng/ml induced a significant increase in enclosing radius and maximum number of intersections compared to controls without GF supplementation (Fig 4, S1, S2 and S4 Figs, S1–S3 Files). While this effect was also observed with 150ng/ml GDNF at the first day of culture, it was abolished at day 3 and 7. Increased maximum numbers of intersections were also observed when compared to IGF treated cultures from day 3 on, whereas enclosing radius and radius of maximum intersections were similar between GDNF and IGF treated cultures. The positive effect of GDNF on neurite outgrowth and arborisation was also observed in combination with other growth factors (COMBO3).

Although IGF supplementation caused a slight increase in the maximum number of intersections at day 1 compared to non-supplemented controls, this effect vanished from day 3 on onwards, also when compared to cultures supplemented with other exogenously added growth factors (GDNF, NGF and combined formula) (Fig 4, S1 File). In contrast, IGF supplementation increased the neurite length (S2 Fig, S3 File). This effect was clearest at day 1, where differences were significant to non-supplemented cultures and the other two growth factors. These results were in line with microscopical observations of long single processes or few processes in IGF supplemented neurons with scarce ramification rather than an extensive arborized network of neurites (S4 Fig).

NGF supplementation had a positive effect on the number of intersections, however, this was most evident after day 3 in culture (Fig 4, S1 File). A positive effect on neurite length was also observed on day 3, whereas at days 1 and 7 no clear effect was detected. Unexpectedly, at day 7 of culture in medium containing 50 ng/ml NGF, neurons exhibited a lower number and shorter neurites compared to neurons maintained in the presence of lower and higher NGF concentrations. NGF supplementation had no effect on the maximum radius of intersections (S1 Fig, S2 File).

## Discussion

DRG cultures have been developed from embryonic, postnatal, or adult rats, mice, rabbits, chickens, carnivores and primates [5, 29]. This study describes the establishment of dissociated primary DRG cultures from calves, which represents a sustainable host-specific model due to the fact that DRGs represent waste products of the regular slaughter process for food consumption. Bovine postnatal DRG cultures can be prepared on a regular basis due to the availability of tissues from slaughtered animals, and are not dependent on the availability of experimental animals. Thus, this culture technique contributes to the internationally accepted 3Rs principle of replacement, reduction, and refinement in animal experimentation [30].

Isolation of bovine postnatal DRG is easy, rapid and takes advantage of the slaughtering process as the siding of the carcass in two halves exposes the neuroforamina within a few minutes, allowing for the collection of DRGs without the need for complex anatomical preparations. It is possible to collect at least 20 DRGs from a single animal in less than 15 minutes with a yield similar to DRG dissociations of postnatal laboratory animals [5, 12, 17, 31]. In contrast, the yield from embryonal DRG of laboratory animals is generally superior [12, 29]. Compared to embryonal cultures, however, postnatal cultures more closely reflect DRG physiology by providing a source of mature neurons and, hence, are more suitable to study acquired pathologies [2, 29].

The current study provides a method to produce viable cells from slaughtered animals, which are amenable to biochemical and electrophysiological studies. With soma diameters ranging from 20 to 200 μm postnatal DRG sensory neurons are large when compared to other species [32–35]. This fact could be explained by the reported correlation of neuronal soma size and body size of the animal species [34]. Large neuronal dimensions are exploitable for morphological studies and infection assays facilitating direct injection of pathogens to investigate their ability to replicate intracellularly and to travel intra-axonally. Bovine postnatal DRGs contain a morphologically heterogeneous population of neurons based on the soma size and the presence of neurites. Variation in soma size has also been observed in DRG neurons of other species [34–37] and is related to differences in physiological functions including sensory, metabolic and conductive properties of neurons [36–38]. Whether the morphological heterogeneity observed in bovine DRG neurons reflects biochemical and physiological differences between neuronal subpopulations remains to be determined. Additionally, as we have pooled together DRGs from the cervical, thoracic and lumbar spine for our cultures, we cannot make conclusions about topographical variations in neuronal subpopulations between DRGs of different locations. These have been previously indicated in rodents and ruminants [39, 40].

Medium supplementation with various growth factors had no effect on neuronal viability. This observation is in line with previous studies showing that post-natal and adult neurons do not require exogenously added growth factors for survival [17], whilst embryonal neurons depend on neurotrophic growth factor supplementation for survival in the first week post seeding [29, 31, 41, 42]. Bovine postnatal DRG neurons survived with and without growth factor supplementation for more than 3 weeks in culture, which is similar to primary DRG neuron cultures from other species [5, 17, 31].

Additionally, growth factor supplementation had no effect on the proportion of neurite bearing neurons independently of the neuronal marker used for neurite labeling, which is in contrast to previous studies that observed an increase of neurite bearing rat DRG neurons in NGF supplemented cultures [31, 41]. Independently of the growth factors used, a similar proportion of DRG neurons established an intricate network of neurites that were in close contact with Schwann cells. Additionally, in neurite bearing bovine DRG neurons, extensive neurite outgrowth was present also in medium devoid of added growth factors. These results suggest autocrine or paracrine production of neurotrophic factors by the DRG neurons themselves [43] or by contaminating Schwann cells, satellite cells and fibroblasts [44–46]. Indeed, DRG cultures contained non-neuronal cells, most of which were closely attached to the DRG neurons similar to the anatomical arrangement of satellite cells around neurons *in vivo* and which tended to become confluent by day 7 in culture. However, complete removal of non-neuronal cells was impossible as indicated also by studies in other species [47]. The amount of non-neuronal cells in these cultures may explain absence of any effect of NGF on the number of neurite bearing neurons compared to studies in other animal species [31, 41, 48]. Additionally, the type of NGF used may explain the differences to the aforementioned studies [31, 41, 49]. However, additional species-related differences in physiology cannot be excluded. Experiments with cultures at low cell densities including control conditions with blocking antibodies against growth factors would be required to clarify this question.

Non-neuronal cells that were attached to neurons expressed S100, GFAP, indicating Schwann and satellite cell origin and E-cadherin and Vimentin [3, 47, 50, 51]. The presence of these cells allows for the examination of peripheral neuron-glial interactions during non-infectious and infectious nerve injury and pain [3, 14, 47, 52–54]. Periaxin, a protein involved in peripheral nerve myelination was not expressed by these cells and additionally, IF analysis and transmission electron microscopy did not reveal neurites enwrapped by Schwann cell myelin sheaths. These results suggest that Schwann cells in our cultures were non-myelinating [55]. However, satellite cell processes were frequently observed to enclose neurites.

The population of neurons that emitted neurites generally showed multipolar morphology, distinct from the typical pseudo-unipolar morphology of DRG neurons *in vivo* [56]. Loss of pseudounipolarity has been described also in cultured DRG neurons from rat, mice and rabbit and have been attributed to different factors including the removal of satellite cells, which prevent dendritic formation *in vivo* [8], the type of GF supplementation [57] and activation of transcription factors associated to the regenerative state of dissociated neurons [58]. As in DRG cultures from other species [47, 59] postnatal bovine DRG neurons still had significant numbers of ensheathing cells attached, which does not support the removal of satellite cells as the cause for multipolarity. In contrast, neurite outgrowth, arborisation and elongation were modulated under certain GF supplementations suggesting that presence of GF is involved in neuronal morphology and loss of neuronal pseudounipolarity.

In line with previous studies performed in other species [7, 60], the presence of GDNF significantly propagated neurite outgrowth in terms of number and length of neurites compared to controls without GF supplementation. However, at the highest concentration, this effect was abolished from day 3 on. While the cause for the absent stimulation of neurite outgrowth at this concentration at later time points remains unknown, the lacking effect coincides with the increase of the non-neuronal cell population. Hence, decrease of neurite outgrowth could be explained by endogenous GDNF production of glial cells potentially resulting in inhibitory effects of GDNF at high concentrations [61, 62]. In contrast, the main effect of IGF supplementation was increased neurite length compared to non-supplemented controls and decrease of neurite arborisation compared to GDNF supplementation. The morphology of IGF supplemented neurons frequently differed to that of GDNF supplemented neurons as they assumed a rather pseudo-unipolar like morphology with a single or few long and scarcely ramified processes. These results suggest that IGF stimulates axonal development rather than extensive neurite arborisation supporting *in vivo* observations that IGF inhibits the arborisation of sensory neurons and promotes the elongation of neurites [63]. In line with studies in other species [7, 60], NGF supplementation promoted neurite length and arborisation. This effect was clear at day 3, but not consistently present at the other time points, notably at a concentration of 50ng/ml. The cause for this inconsistency remains unclear. Similarly, neurons supplemented either with a combination of NGF and GDNF or a combination of NGF, GDNF and IGF promoted increase in number and length of neurites compared to controls, but no synergistic effect between GF was observed. This study was restricted to the morphological effect of growth factors on neurons, neurite outgrowth and branching. In order to investigate effects of GF supplementation on the neurochemical phenotype of neurons, as it has been demonstrated in rats, mice and chicken [39, 62, 64], more in-depth studies including the analysis of neuropeptide expression are required.

The analysis of neurite branching revealed that minimum and maximum branching were correlated, independently of the growth factor supplementation. As a general rule, the dendritic field and branching patterns of a neuron are determined by its physiological role. However the balance between metabolic costs of new neurite formation and the need to cover the neuronal receptive field presumably contributes to neurite morphology [65], which is–once established—maintained along the neuronal lifespan [66]. Although the present study investigated *in vitro* cultures of neurons in absence of target tissue as seen *in vivo*, our results show that the number of branching neurites is proportional to the number of non-dividing terminal branches produced by a neuron at any time point indicating that branching activity remains constant during neuronal lifespan, which is in line with previous studies in other species [65, 67, 68]. Thus, post-natal bovine DRG neurons appear to be suitable for studies investigating the mechanism of neuronal outgrowth and regeneration.

## Conclusion

We describe a method for the establishment of a primary postnatal bovine DRG neuron culture from slaughtered calves, which can be maintained for extended periods of time. We assessed the neurite growth potential of dissociated bovine post-natal sensory neurons *in vitro* and showed that growth factor supplementation is not required for their survival but modulates neurite outgrowth. Furthermore, this mixed cell culture recapitulates the organizational architecture between neurons, neurites and ensheathing cells in the peripheral nervous system. Therefore, this system represents a sustainable host-specific model for neuroscience research and may be used in dynamic growth assays, pharmacological and toxicological screenings, for studies on neurite regeneration, pain and pathomechanisms of diseases involving the peripheral nervous system.

## Supporting Information

S1 Fig Radius of maximum number of intersection in DRG neurons at day 1 (A), day 3 (B) and day 7 (C) in culture. Box-and-whisker plots (5–95 percentiles) show the radius of maximum of intersections (NGF: 10, 50, 150 ng/ml; GDNF: 10, 50, 150 ng/ml; IGF: 5, 20, 50 ng/ml, NGF 50 ng/ml combined with GDNF 50 ng/ml [COMBO2]; NGF 50ng/ml combined with GDNF 50 ng/ml and IGF20 ng/ml [COMBO3]) and control (No GF). Values below and above the whiskers are shown as individual points. Horizontal black lines indicate the median. Ninety neurons were analyzed for each GF supplementation group and control group. (A) At day 1, the radius at the maximum of intersection was significantly higher in neurons supplemented with GDNF50, IGF20, IGF50, NGF50 and with a combination of NGF 50 ng/ml, GDNF 50 ng/ml and IGF 20 ng/ml [COMBO3] than in the control. (B-C) From day 3 to day 7, differences between supplementations were no longer statistically significant. (D) Comparison of radius of maximum number of intersections over time between groups of supplementation and control. The radius of maximum number of intersections increases in all groups from day 1 to day 7 in culture. Mean values of the radius of maximum number of intersections calculated with the Sholl analysis are shown. Results of the statistical analysis are shown in S2 File.
(TIF)

S2 Fig Enclosing radius in DRG neurons at day 1 (A), day 3 (B) and day 7 (C) in culture. Box-and-whisker plots (5–95 percentiles) show the enclosing radius of groups of supplementation (NGF: 10, 50, 150 ng/ml; GDNF: 10, 50, 150 ng/ml; IGF: 5, 20, 50 ng/ml, NGF 50 ng/ml combined with GDNF 50 ng/ml [COMBO2]; NGF 50ng/ml combined with GDNF 50 ng/ml and IGF20 ng/ml [COMBO3]) and control (No GF). Values below and above the whiskers are shown as individual points. Horizontal black lines indicate the median. Ninety neurons were analyzed for each GF supplementation and control group. At day 1, the enclosing radius was significantly bigger in neurons supplemented with GDNF150, IGF20 or IGF50 compared to the control. (B) At day 3, the enclosing radius of neurons supplemented with GDNF10, IGF5, IGF20, IGF50, NGF10, NGF150, or with a combination of growth factors including IGF (COMBO3) or just NGF 50 and GDNF 50 (COMBO2) was significantly increased compared to control neurons. (C) At day 7 the enclosing radius of neurons supplemented with GDNF50 and a combination of growth factors including IGF (COMBO3) was significantly bigger than that of control neurons, while neurons supplemented with GDNF150, IGF5 and NGF 50 showed a significantly lower value of enclosing radius. (D) Comparison of enclosing radius over time between groups of supplementation and control. The enclosing radius increases in all groups from day 1 to day 7 in culture. Mean values of the enclosing radius calculated with the Sholl analysis are shown. Results of the statistical analysis are shown in S3 File.
(TIF)

S3 Fig Double immunofluorescence of dissociated neurons at day 3 of culture. Neurons are labeled with anti-NF (green, A) and (B) anti-βIII tubulin (red, B). (C) Merged image showing colocalization of NF and βIII tubulin both in neurons with and without neurites.
(TIF)

S4 Fig Immunofluorescence of DRG neurons (Day 7). Neurons are supplemented with GDNF (A), IGF (B) or cultured in absence of GF supplementation (control, C). (A) The presence of GDNF increases neurite outgrowth compared to control. (B) IGF medium supplementation increases the number of neurons bearing few long and scarcely ramified processes. (C) In neurite bearing neurons, extensive neurite outgrowth is present also in absence of GF supplementation. DRG neurons are stained with anti-NF antibody (grey).
(TIF)

S5 FigCorrelation Scatter XY plots showing the means of minimum ($\bar{Bmin^{tr}}$) and maximum ($\bar{Bmax^{tr}}$) branching rate at day 1 (A), day 3 (B) and day 7 (C).Minimum and maximum branching rates are highly correlated, and correlation between minimum and maximum branching remains constant over time. Each different color represents a different GF supplementation and control (No GF). Pearson's correlation coefficients were -0.532 (day 1), -0.66 (day 3) and -0.56 (day 7).(TIF)

S6 Fig Transmission electron microscopy of dissociated DRG in culture. (A) Overview of non-myelinating Schwann cells enclosing a neurite (white box). Bar = 1100 nm. (B) Higher magnification of the boxed frame in A. The longitudinally sectioned neurite contains neurofilaments and microtubules. The neurite is encircled by a Schwann cell without myelin sheath. Bar = 250 nm. (C) Two adjacent neurons (N) are separated by two flattened Satellite cells that are closely surrounding the upper neuron. The Satellite cells contain a large number of membrane-bound vacuoles and have numerous elongated cytoplasmic processes. Bar = 1100 nm (D) High magnification of a Satellite cell showing cilia (Ci), one extruding into the extracellular space, and the associated ciliary pockets (CiP). Bar = 250 nm.
(TIF)

S1 File Statistical analysis of “maximum of intersections” data. Kruskal-Wallis statistic with Dunn's Multiple Comparison Test, threshold significance level for P is 0.05. P value < 0.001 is considered extremely significant (***); P value from 0.001 to 0.01 very significant (**); P value from 0.01 to 0.05 significant (*); P value > 0.05 not significant (ns).
(XLSX)

S2 File Statistical analysis of “radius of maximum of intersections” data. Kruskal-Wallis statistic with Dunn's Multiple Comparison Test, threshold significance level for P is 0.05. P value < 0.001 is considered extremely significant (***); P value from 0.001 to 0.01 very significant (**); P value from 0.01 to 0.05 significant (*); P value >0.05 not significant (ns).
(XLSX)

S3 File Statistical analysis of “enclosing radius” data. Kruskal-Wallis statistic with Dunn's Multiple Comparison Test, threshold significance level for P is 0.05. P value < 0.001 is considered extremely significant (***); P value from 0.001 to 0.01 very significant (**); P value from 0.01 to 0.05 significant (*); P value >0.05 not significant (ns).
(XLSX)

## References

[1] Pannese E. The structure of the perineuronal sheath of satellite glial cells (SGCs) in sensory ganglia. Neuron Glia Biol. 2010;6(1):3–10. 10.1017/S1740925X10000037 20604977

[2] Huang LY, Gu Y, Chen Y. Communication between neuronal somata and satellite glial cells in sensory ganglia. Glia. 2013;61(10):1571–81. 10.1002/glia.22541 23918214 PMC3758405

[3] Hanani M. Satellite glial cells in sensory ganglia: from form to function. Brain Res Brain Res Rev. 2005;48(3):457–76. 10.1016/j.brainresrev.2004.09.001 15914252

[4] Mirsky R, Jessen KR, Brennan A, Parkinson D, Dong Z, Meier C, et al Schwann cells as regulators of nerve development. J Physiol. 2002;96(1–2):17–24. 10.1016/s0928-4257(01)00076-6 11755779

[5] Malin SA, Davis BM, Molliver DC. Production of dissociated sensory neuron cultures and considerations for their use in studying neuronal function and plasticity. Nat Protoc. 2007;2(1):152–60. 10.1038/nprot.2006.461 17401349

[6] Curanovic D, Ch'ng TH, Szpara M, Enquist L. Compartmented neuron cultures for directional infection by alpha herpesviruses. Curr Protoc Cell Biol. 2009;Chapter 26(Unit 26.4):Unit 26 4. 10.1002/0471143030.cb2604s43 PMC2718536 19499506

[7] Wong AW, KPY J, Payne SC, Keast JR, Osborne PB. Neurite outgrowth in normal and injured primary sensory neurons reveals different regulation by nerve growth factor (NGF) and artemin. Mol Cell Neurosci. 2015;65:125–34. Epub Epub 2015 Mar 6. 10.1016/j.mcn.2015.03.004 25752731

[8] De Koninck P, Carbonetto S, Cooper E. NGF induces neonatal rat sensory neurons to extend dendrites in culture after removal of satellite cells. J Neurosci. 1993;13(2):577–85. 8426228 10.1523/JNEUROSCI.13-02-00577.1993 PMC6576645

[9] Kalous A, Keast JR. Conditioning lesions enhance growth state only in sensory neurons lacking calcitonin gene-related peptide and isolectin B4-binding. Neuroscience. 2010;166(1):107–21. Epub Epub 2009 Dec 16. 10.1016/j.neuroscience.2009.12.019 20006678

[10] Muratori L, Ronchi G, Raimondo S, Geuna S, Giacobini-Robecchi MG, Fornaro M. Generation of new neurons in dorsal root ganglia in adult rats after peripheral nerve crush injury. Neural Plast. 2015;2015:860546 10.1155/2015/860546 25722894 PMC4333329

[11] Bauer A, Nolden T, Schroter J, Romer-Oberdorfer A, Gluska S, Perlson E, et al Anterograde glycoprotein-dependent transport of newly generated rabies virus in dorsal root ganglion neurons. J Virol. 2014;88(24):14172–83. 10.1128/JVI.02254-14 25275124 PMC4249153

[12] Owen DE, Egerton J. Culture of dissociated sensory neurons from dorsal root ganglia of postnatal and adult rats. Methods Mol Biol. 2012;846:179–87. 10.1007/978-1-61779-536-7_16 22367811

[13] Chowdhury SI, Brum MC, Coats C, Doster A, Wei H, Jones C. The bovine herpesvirus type 1 envelope protein Us9 acidic domain is crucial for anterograde axonal transport. Vet Microbiol. 2011;152(3–4):270–9. 10.1016/j.vetmic.2011.05.012 21640524

[14] Huang T, Cherkas P, Rosenthal D. Dye coupling among satellite glial cells in mammalian dorsal root ganglia. Brain Res Brain Res Rev. 2005;1036(1–2):42–9. 10.1016/j.brainres.2004.12.021 15725400

[15] Merighi A, Kar S, Gibson SJ, Ghidella S, Gobetto A, Peirone SM, et al The immunocytochemical distribution of seven peptides in the spinal cord and dorsal root ganglia of horse and pig. Anat Embryol. 1990;181(3):271–80. 1692451 10.1007/BF00174620

[16] Kobayashi S, Kokubo Y, Uchida K, Yayama T, Takeno K, Negoro K, et al Effect of lumbar nerve root compression on primary sensory neurons and their central branches: changes in the nociceptive neuropeptides substance P and somatostatin. Spine (Phila Pa 1976) 2005;30(3):276–82. 15682006 10.1097/01.brs.0000152377.72468.f4

[17] Gerhauser I, Hahn K, Baumgartner W, Wewetzer K. Culturing adult canine sensory neurons to optimise neural repair. Vet Rec. 2012;170(4):102. Epub 2011 Nov 8. 10.1136/vr.100255 22068333

[18] Morgan BR, Coates JR, Johnson GC, Shelton GD, Katz ML. Characterization of thoracic motor and sensory neurons and spinal nerve roots in canine degenerative myelopathy, a potential disease model of amyotrophic lateral sclerosis. J Neurosci Res. 2014;92(4):531–41. 10.1002/jnr.23332 24375814 PMC4096142

[19] Pannese E, Ventura R, Bianchi R. Quantitative relationships between nerve and satellite cells in spinal ganglia: an electron microscopical study. II. Reptiles. J Comp Neurol. 1975;160(4):463–76. 10.1002/cne.901600404 1123463

[20] Yamashita S. Heat-induced antigen retrieval: mechanisms and application to histochemistry. Prog Histochem Cytochem. 2007;41(3):141–200. 10.1016/j.proghi.2006.09.001 17197287

[21] Hemphill A, Croft SL. Electron Microscopy in Parasitology In: Rogan MT, editor. Analytical Parasitology. Berlin, Heidelberg: Springer Berlin Heidelberg; 1997 p. 227–68.

[22] Winzer P, Muller J, Aguado-Martinez A, Rahman M, Balmer V, Manser V, et al In Vitro and In Vivo Effects of the Bumped Kinase Inhibitor 1294 in the Related Cyst-Forming Apicomplexans Toxoplasma gondii and Neospora caninum. Antimicrob Agents Chemother. 2015;59(10):6361–74. 10.1128/AAC.01236-15 26248379 PMC4576114

[23] Sholl DA. Dendritic organization in the neurons of the visual and motor cortices of the cat. J Anat. 1953;87(4):387–406. 13117757 PMC1244622

[24] Ferreira TA, Blackman AV, Oyrer J, Jayabal S, Chung AJ, Watt AJ, et al Neuronal morphometry directly from bitmap images. Nat Methods. 2014;11(10):982–4. 10.1038/nmeth.3125 25264773 PMC5271921

[25] Hatzig SV, Schiessl S, Stahl A, Snowdon RJ. Characterizing root response phenotypes by neural network analysis. J Exp Bot. 2015;66(18):5617–24. 10.1093/jxb/erv235 26019255 PMC4585416

[26] Benmerah A. The ciliary pocket. Curr Opin Cell Biol. 2013;25(1):78–84. 10.1016/j.ceb.2012.10.011 23153502

[27] Voigt T, Dauber W, Kohler U. Perisynaptic Schwann cells of the vertebrate motor endplate bear modified cilia. Microsc Res Tech 2004 2 15;63(3):149–54. 10.1002/jemt.20023 14755601

[28] Grillo MA, Palay SL. Ciliated Schwann cells in the autonomic nervous system of the adult rat. J Cell Biol. 1963;16(Feb):430–6. 13950483 10.1083/jcb.16.2.430 PMC2106254

[29] Melli G, Hoke A. Dorsal Root Ganglia Sensory Neuronal Cultures: a tool for drug discovery for peripheral neuropathies. Expert Opin Drug Discov. 2009;4(10):1035–45. 10.1517/17460440903266829 20657751 PMC2908326

[30] Franco NH, Olsson IA. Scientists and the 3Rs: attitudes to animal use in biomedical research and the effect of mandatory training in laboratory animal science. Lab Anim. 2014;48(1):50–60. 10.1177/0023677213498717 23940123

[31] Lindsay R. Nerve growth factors (NGF, BDNF) enhance axonal regeneration but are not required for survival of adult sensory neurons. J Neurosci. 1988;8(7):2394–405. 3249232 10.1523/JNEUROSCI.08-07-02394.1988 PMC6569525

[32] Price J. An immunohistochemical and quantitative examination of dorsal root ganglion neuronal subpopulations. J Neurosci. 1985;5(8):2051–9. 2410579 10.1523/JNEUROSCI.05-08-02051.1985 PMC6565294

[33] Barabas ME, Mattson EC, Aboualizadeh E, Hirschmugl CJ, Stucky CL. Chemical structure and morphology of dorsal root ganglion neurons from naive and inflamed mice. J Biol Chem. 2014;289(49):34241–9. 10.1074/jbc.M114.570101 25271163 PMC4256355

[34] Khan AA, Dilkash MNA, Khan MA, Faruqi NA. Morphologically atypical cervical dorsal root ganglion neurons in adult rabbit. Biomedical Research. 2009;20(1):45–9.

[35] Pierce LM, Rankin MR, Foster RT, Dolber PC, Coates KW, Kuehl TJ, et al Distribution and immunohistochemical characterization of primary afferent neurons innervating the levator ani muscle of the female squirrel monkey. Am J Obstet Gynecol. 2006;195(4):987–96. Epub 2006 Apr 25. 10.1016/j.ajog.2006.02.042 16635454

[36] Averill S, McMahon SB, Clary DO, Reichardt LF, Priestley JV. Immunocytochemical localization of trkA receptors in chemically identified subgroups of adult rat sensory neurons. Eur J Neurosci. 1995;7(7):1484–94. 7551174 10.1111/j.1460-9568.1995.tb01143.x PMC2758238

[37] Usoskin D, Furlan A, Islam S, Abdo H, Lonnerberg P, Lou D, et al Unbiased classification of sensory neuron types by large-scale single-cell RNA sequencing. Nat Neurosci. 2015;18(1):145–53. 10.1038/nn.3881 25420068

[38] Harper AA, Lawson SN. Conduction velocity is related to morphological cell type in rat dorsal root ganglion neurones. J Physiol 1985;359:31–46. 3999040 10.1113/jphysiol.1985.sp015573 PMC1193363

[39] Price TJ, Flores CM. Critical evaluation of the colocalization between calcitonin gene-related peptide, substance P, transient receptor potential vanilloid subfamily type 1 immunoreactivities, and isolectin B4 binding in primary afferent neurons of the rat and mouse. J Pain. 2007 8(3):263–72. 10.1016/j.jpain.2006.09.005 17113352 PMC1899162

[40] Russo D, Clavenzani P, Mazzoni M, Chiocchetti R, Di Guardo G, Lalatta-Costerbosa G. Immunohistochemical characterization of TH13-L2 spinal ganglia neurons in sheep (Ovis aries). Microsc Res Tech. 2010;73(2):128–39. 10.1002/jemt.20764 19725058

[41] Gavazzi I, Kumar RD, McMahon SB, Cohen J. Growth responses of different subpopulations of adult sensory neurons to neurotrophic factors in vitro. Eur J Neurosci. 1999;11(10):3405–14. 10564348 10.1046/j.1460-9568.1999.00756.x

[42] Tong JX, Eichler ME, Rich KM. Intracellular calcium levels influence apoptosis in mature sensory neurons after trophic factor deprivation. Exp Neurol. 1996;138(1):45–52. 10.1006/exnr.1996.0045 8593895

[43] Harrington AW, Ginty DD. Long-distance retrograde neurotrophic factor signalling in neurons. Nat Rev Neurosci. 2013;14(3):177–87. 10.1038/nrn3253 23422909

[44] Hanani M. Satellite glial cells in sensory ganglia: from form to function. Brain Res Brain Res Rev. 2005;48(3):457–76. 10.1016/j.brainresrev.2004.09.001 15914252

[45] Matsuoka I, Meyer M, Thoenen H. Cell-type-specific regulation of nerve growth factor (NGF) synthesis in non-neuronal cells: comparison of Schwann cells with other cell types. J Neurosci. 1991;11(10):3165–77. 1658245 10.1523/JNEUROSCI.11-10-03165.1991 PMC6575453

[46] Arce V, Pollock RA, Philippe JM, Pennica D, Henderson CE, deLapeyriere O. Synergistic effects of schwann- and muscle-derived factors on motoneuron survival involve GDNF and cardiotrophin-1 (CT-1). J Neurosci. 1998;18(4):1440–8. 9454853 10.1523/JNEUROSCI.18-04-01440.1998 PMC6792716

[47] Christie K, Koshy D, Cheng C, Guo G, Martinez JA, Duraikannu A, et al Intraganglionic interactions between satellite cells and adult sensory neurons. Mol Cell Neurosci. 2015;67:1–12. 10.1016/j.mcn.2015.05.001 25979201

[48] Madduri S, Papaloizos M, Gander B. Synergistic effect of GDNF and NGF on axonal branching and elongation in vitro. Neurosci Res. 2009;65(1):88–97. 10.1016/j.neures.2009.06.003 19523996

[49] Colangelo AM, Finotti N, Ceriani M, Alberghina L, Martegani E, Aloe L, et al Recombinant human nerve growth factor with a marked activity in vitro and in vivo. Proc Natl Acad Sci U S A. 2005;102(51):18658–63. 10.1073/pnas.0508734102 16339317 PMC1317951

[50] Madarame H, Seuberlich T, Abril C, Zurbriggen A, Vandevelde M, Oevermann A. The distribution of E-cadherin expression in listeric rhombencephalitis of ruminants indicates its involvement in Listeria monocytogenes neuroinvasion. Neuropathol Appl Neurobiol. 2011;37(7):753–67. 10.1111/j.1365-2990.2011.01183.x 21486315

[51] Shimamura K, Takahashi T, Takeichi M. E-cadherin expression in a particular subset of sensory neurons. Dev Biol. 1992;152(2):242–54. 1644219 10.1016/0012-1606(92)90132-z

[52] Nascimento RS, Santiago MF, Marques SA, Allodi S, Martinez AM. Diversity among satellite glial cells in dorsal root ganglia of the rat. Braz J Med Biol Res. 2008;41(11):1011–7. 19030716 10.1590/s0100-879x2008005000051

[53] Huang LY, Gu Y, and Chen Y. Communication between neuronal somata and satellite glial cells in sensory ganglia. Glia. 2013;61(10):1571–81. 10.1002/glia.22541 23918214 PMC3758405

[54] Kushnir R, Cherkas PS, Hanani M. Peripheral inflammation upregulates P2X receptor expression in satellite glial cells of mouse trigeminal ganglia: a calcium imaging study. Neuropharmacology. 2011;61(4):739–46. 10.1016/j.neuropharm.2011.05.019 21645532

[55] Crawford AT, Desai D, Gokina P, Basak S, Kim HA. E-Cadherin Expression in Postnatal Schwann Cells Is Regulated by the cAMP-Dependent Protein Kinase A Pathway. Glia. 2008;56(15):1637–47. 10.1002/glia.20716 18551621 PMC2575062

[56] De Lahunta A, Glass EN, Kent M. Veterinary Neuroanatomy and Clinical Neurology, 4th Edition 2015.

[57] Dupraz S, Grassi D, Karnas D, Nieto Guil AF, Hicks D, Quiroga S. The insulin-like growth factor 1 receptor is essential for axonal regeneration in adult central nervous system neurons. PLoS One. 2013;8(1):e54462 10.1371/journal.pone.0054462 23349896 PMC3548777

[58] Frey E, Valakh V, Karney-Grobe S, Shi Y, Milbrandt J, DiAntonio A. An in vitro assay to study induction of the regenerative state in sensory neurons. Exp Neurol. 2015;263:350–63. 10.1016/j.expneurol.2014.10.012 25447942 PMC4266464

[59] Valtcheva MV, Copits BA, Davidson S, Sheahan TD, Pullen MY, McCall JG, et al Surgical extraction of human dorsal root ganglia from organ donors and preparation of primary sensory neuron cultures. Nat Protoc. 2016;11(10):1877–88. 10.1038/nprot.2016.111 27606776 PMC5082842

[60] Paveliev M, Airaksinen MS, Saarma M. GDNF family ligands activate multiple events during axonal growth in mature sensory neurons. Mol Cell Neurosci. 2004;25(3):453–9. 10.1016/j.mcn.2003.11.010 15033173

[61] Deister C, Schmidt CE. Optimizing neurotrophic factor combinations for neurite outgrowth. J Neural Eng. 2006;3(2):172–9. 10.1088/1741-2560/3/2/011 16705273

[62] Mills CD, Allchorne AJ, Griffin RS, Woolf CJ, Costigan M. GDNF selectively promotes regeneration of injury-primed sensory neurons in the lesioned spinal cord. Mol Cell Neurosci. 2007;36(2):185–94. 10.1016/j.mcn.2007.06.011 17702601 PMC2034440

[63] Scolnick JA, Cui K, Duggan CD, Xuan S, Yuan XB, Efstratiadis A, et al Role of IGF signaling in olfactory sensory map formation and axon guidance. Neuron. 2008;57(6):847–57. 10.1016/j.neuron.2008.01.027 18367086 PMC2364597

[64] Forgie A, Doxakis E, Buj-Bello A, Wyatt S, Davies AM. Differences and developmental changes in the responsiveness of PNS neurons to GDNF and neurturin. Mol Cell Neurosci. 1999;13(6):430–40. 10.1006/mcne.1999.0760 10383828

[65] Jan YN, Jan LY. Branching out: mechanisms of dendritic arborization. Nat Rev Neurosci. 2010;11(5):316–28. 10.1038/nrn2836 20404840 PMC3079328

[66] Goldberg J. Intrinsic neuronal regulation of axon and dendrite growth. Curr Opin Neurobiol. 2004;14(5):551–7. 10.1016/j.conb.2004.08.012 15464887

[67] Konishi Y, Stegmuller J, Matsuda T, Bonni S, Bonni A. Cdh1-APC controls axonal growth and patterning in the mammalian brain. Science. 2004;303(5660):1026–30. 10.1126/science.1093712 14716021

[68] Peng YR, He S, Marie H, Zeng SY, Ma J, Tan ZJ, et al Coordinated changes in dendritic arborization and synaptic strength during neural circuit development. Neuron. 2009;61(1):71–84. 10.1016/j.neuron.2008.11.015 19146814 PMC2713111

