# Supplementary figures and images for: Primary Postnatal Dorsal Root Ganglion Culture from Conventionally Slaughtered Calves
Source: PLoS One. 2016 Dec 9;11(12):e0168228. doi: 10.1371/journal.pone.0168228 (PMC5148591; doi:10.1371/journal.pone.0168228)

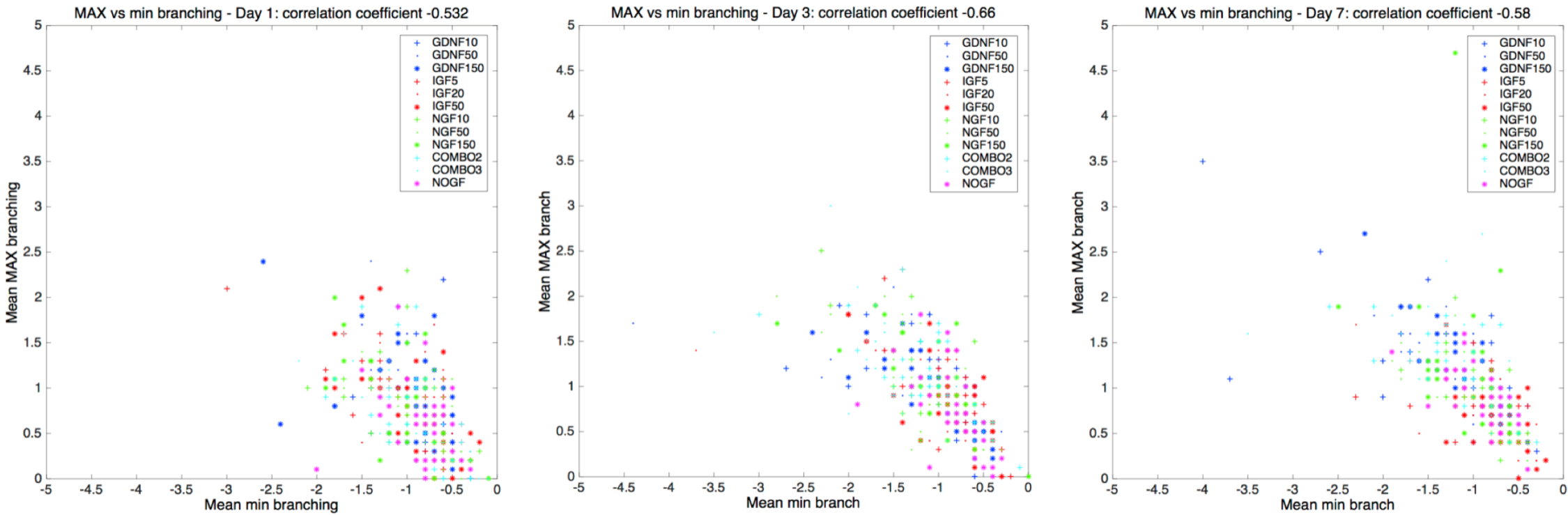

Supplement: S5 Fig — Minimum and maximum branching rates are highly correlated, and correlation between minimum and maximum branching remains constant over time. Each different color represents a different GF supplementation and control (No GF). Pearson's correlation coefficients were -0.532 (day 1), -0.66 (day 3) and -0.56 (day 7). (TIF) [file pone.0168228.s005.tif]
